# Usefulness of uterine artery Doppler velocimetry as a predictor for hypertensive disorders in pregnancy in women with prehypertension before 20 weeks gestation

**DOI:** 10.1371/journal.pone.0210566

**Published:** 2019-01-30

**Authors:** Seung Woo Yang, Soo Hyun Cho, Young Sun Kang, Seung Hwa Park, In Sook Sohn, Han Sung Kwon, Han Sung Hwang

**Affiliations:** 1 Division of Maternal and Fetal Medicine, Department of Obstetrics and Gynecology, Research Institute of Medical Science, Konkuk University School of Medicine, Seoul, Republic of Korea; 2 Department of Anatomy, School of Medicine, Konkuk University, Seoul, Republic of Korea; 3 Department of Biomedical Science & Technology, Konkuk University, Seoul, Republic of Korea; 4 Department of Veterinary Pharmacology and Toxicology, College of Veterinary Medicine, Konkuk University, Seoul, Korea; Seoul National University Bundang Hospital, REPUBLIC OF KOREA

## Abstract

Hypertensive disorders of pregnancy (HDP) is major complication of maternal-fetal outcomes in obstetric field. Although HDP is mainly defined by high blood pressure, the information about the relationship between prehypertension (preHTN, 120-139mmHg and 80-89mmHg) and HDP development is limited. The objective of this study is to determine the usefulness of preHTN before 20 weeks gestation and uterine artery (UtA) Doppler velocimetry as a predictor of HDP. A total of 2039 singleton pregnant women who had received continuous prenatal care were included in this study. The participants were classified into 2 groups based on the highest blood pressure (BP) under 20 gestational weeks as defined by the Joint National Committee 7: Normotensive (n = 1816) and preHTN pregnant women (n = 223). All preHTN pregnant women were assessed using UtA Doppler velocimetry, and the numbers of preHTN assessments were recorded. The risk of HDP was assessed in the PreHTN groups through patient history and Doppler velocimetry. Compared to normotensive patients, a total of 223 preHTN patients had a higher risk of preeclampsia (OR: 2.3; CI: 1.2–4.3), gestational hypertension (OR: 3.3; CI: 2.0–5.4) and any HDP (OR: 3.0; CI: 2.0–4.5). In the preHTN group, 134 (60.1%) patients had preHTN measured at least twice and 89 (39.9%) patients had preHTN. The results showed that two or more preHTN measurements have high sensitivity for predicting HDP (OR: 1.9; CI: 1.0–3.1; sensitivity: 83.8%; specificity: 47.2%). Additionally, the combination of abnormal UtA Doppler velocimetry results and at least two preHTN measurements showed a high accuracy in predicting HDP (OR: 2.9; CI: 1.1–4.1; sensitivity: 67.6%; specificity: 98.4%). In conclusion, close BP monitoring and recording of every preHTN event are important for pregnant women with preHTN history, and UtA Doppler examination in those women during the 2^nd^ trimester can be a further aid in determining the risk of HDP.

## Introduction

Hypertensive disorders of pregnancy (HDP), including gestational hypertension (GHTN) and preeclampsia (PE), are the most common medical complications of pregnancy, affecting 6% to 8% of pregnant women.[1,2] In particular, PE is a systemic syndrome characterized by new-onset hypertension and proteinuria after 20 weeks of gestation and affects 3% to 5% of pregnant women.[2] PE might coexist with intrauterine growth restriction (IUGR), placental abruption, preterm delivery, and increased risk of maternal cardiovascular events later in life.[3] GHTN is characterized by new-onset elevations of BP after 20 weeks of gestation in the absence of accompanying proteinuria. Thus, early prediction of GHTN and PE is important for planning the appropriate monitoring and clinical management strategy that can decrease the incidence of complications from these conditions and save medical costs.

Blood pressure (BP) measurement is an important screening tool used in obstetric care to detect or predict pregnancy hypertensive disorders. Some studies have shown that high BP in the first trimester is associated with an increased risk of developing GHTN and PE.[4–6] Although high BP is closely related to the risk of pregnancy hypertensive diseases, little is known about the relationship between prehypertension (preHTN) and the risk for HDP. preHTN is defined as a systolic BP of 120 mmHg to 139 mmHg and a diastolic pressure of 80 to 89 mmHg.[7] Recent literature suggests that preHTN earlier than 20 weeks of pregnancy is related to adverse obstetrical outcomes.[8,9]

Recent studies showed that a more effective approach for predicting the occurrence of pregnancy hypertensive disease is one that combines maternal history with measurement of various clinical parameters.[10] Uterine artery (UtA) Doppler velocimetry is among the clinical tools widely used in obstetrics and is a useful screening test in women at high risk of PE.[11,12] Poor placentation with deficient remodeling of the spiral arteries is associated with subsequent development of the early onset form of PE as well as IUGR, and other complications. In these abnormal pregnancies, the uteroplacental circulation remains in a state of high resistance, which can be measured noninvasively via the UtA Doppler ultrasound.[6,13,14] Also, some studies suggested that the combination of biophysical profile, including BP and laboratory results with UtA Doppler values, is more useful for predicting the occurrence of PE.[15] Because measurement of BP is the easiest and most time-saving in clinical settings, the combination of BP measurement with UtA Doppler assessment will be a useful method predicting factor for screening pregnant women at risk for PE after 20 weeks gestation.[16] However, there is no assessment about the relation between perHTN and UtA Doppler velocimetry for predicting HDP.

Therefore, this study aimed to determine the usefulness of a preHTN history before 20 weeks gestation and UtA Doppler velocimetry as a predictor of HDP.

## Materials and methods

**Fig 1:**
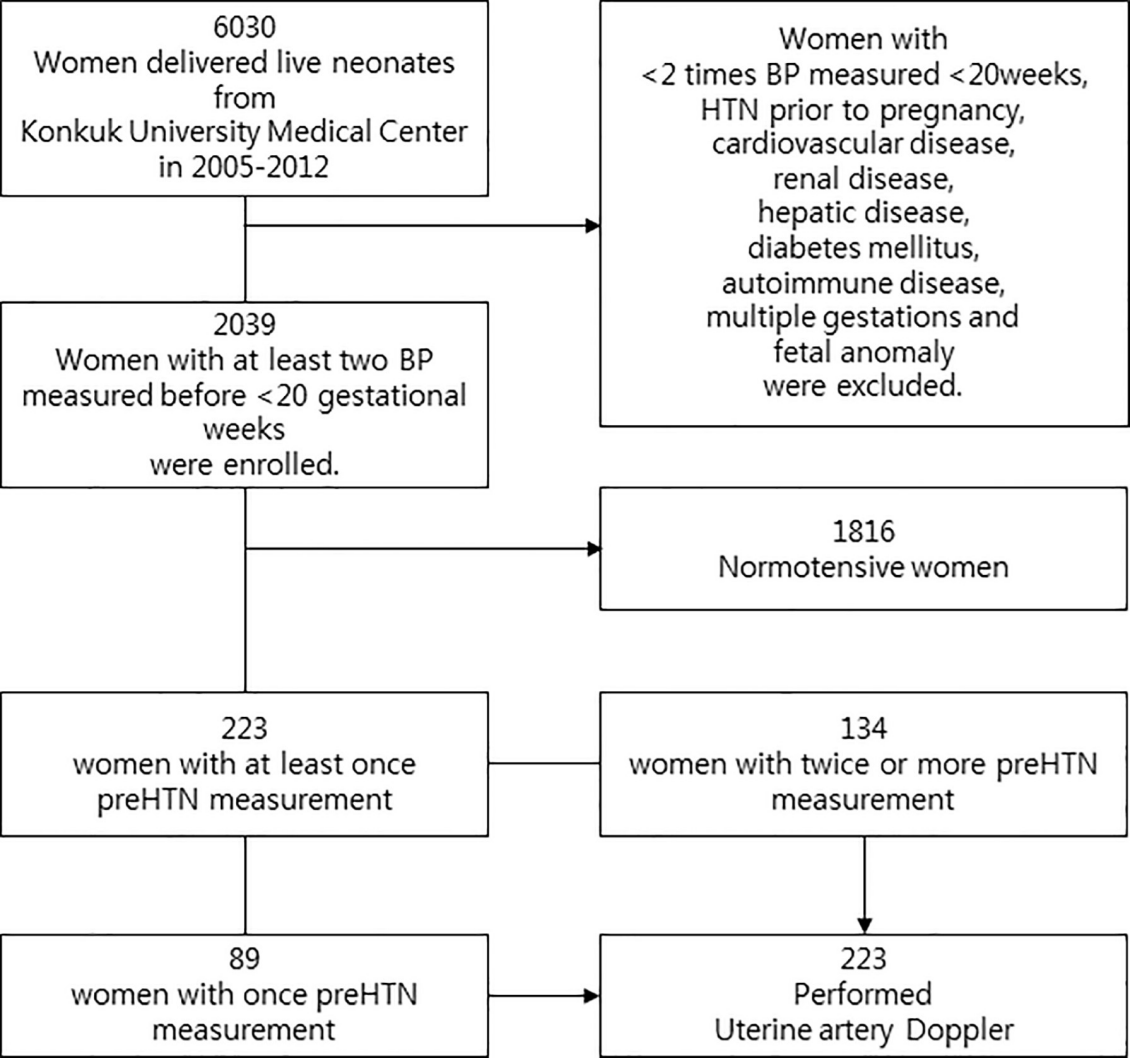
Flow diagram of women and excluded criteria in present study. Total 2039 singleton women who had blood pressure measured >2 times or more before 20 gestational weeks in Konkuk University Medical Center were enrolled. Uterine artery Doppler was performed in all patients in the prehypertension (preHTN) group (n = 223).

Data from all pregnant women who delivered in Konkuk University Medical Center from August 2005 to December 2012 were reviewed. This clinical study approved by Konkuk University Hospital IRB. (IRB number: KUH 1040054) The review board waived the requirement for informed consent for this research. Pregnant women with less than two BP measurements before 20 gestational weeks and those with chronic hypertension, cardiovascular disease, renal disease, hepatic disease, diabetes mellitus, autoimmune disease, multiple gestations, and fetal structural or genetic anomalies were excluded in this study. A total of 2039 singleton pregnant women were enrolled (Fig 1). The BP of all included patients was measured at least twice before 20 gestational weeks using an automatic BP monitor device based on the cuff-oscillometric method (JAWON MEDICAL CO. Korea, model FT-500R PLUS.). For every hospital visit, the patient’s BP was checked in the left arm with the patient in a sitting position after resting for 5 minutes if needed. The patients were divided into two groups according to the Joint National Committee’s 7 criteria as follows: normotensive [systolic BP (SBP) <120 mmHg and diastolic BP (DBP) <80 mmHg] and preHTN (SBP from 120 mmHg to 139 mmHg and DBP from 80 mmHg to 89 mmHg). In the preHTN group, those with preHTN on at least two consecutive ambulatory visits were identified.[7] Patients in the preHTN group were also assessed using the UtA Doppler velocimetry at 21 weeks of gestation via transabdominal ultrasound with the patient in supine position. Abnormal UtA Doppler results was defined as a pulsatility index (PI; calculated as peak systolic velocity-end diastolic velocity/mean velocity) higher than 1.30 (95^th^ percentile).[17–19] The clinical outcomes of patients, including the occurrence of PE and GHTN were measured using the guidelines described by the American College of Obstetricians and Gynecologists. Data were evaluated and compared between the normal and preHTN groups using student’s t-test, χ^2^ test for clinical outcome and logistic regression analysis for odds ratio, performance of preHTN combined with abnormal UtA Doppler, respectively. Data analysis was performed using the Statistical Package for Social Sciences for Windows, version 18.0 (SPSS Inc., Chicago, IL, USA). A p value of <0.05 was considered statistically significant.

## Results

The clinical characteristics of the patients in each group are given in Table 1. Compared with patients in the normal group, those in the preHTN group delivered earlier, had higher BP before 20 weeks gestation and at delivery, their neonates had lower birth weight. In the preHTN group, there were 2 cases of IUGR (defined as 10^th^ percentile per gestational age) were in PE group, but there were no IUGR cases in the GHTN group. Also, the preHTN group had statistically higher risk for HDP, PE and GHTN than did the normal group.

**Table 1:** Clinical characteristics in normal and prehypertensive (preHTN) groups.

	Normal (n = 1,816)	preHTN (n = 223)	p-Value	OR (95% CI)
Maternal age (years)	31.6 ± 2.9	32.1 ± 3.8	0.156	
BMI (kg/m^2^)	21.16±2.5	22.58±3.1	0.092	
Nulliparity (n)	880 (48.5)	148 (66.4)	0.031^**†**^	
Delivery weeks (weeks)	38.1 ± 2.2	36.4 ± 2.1	< 0.001*	
Preeclampsia		35.2± 3.4		
Gestational hypertension		37.8± 2.3		
Systolic blood pressure at delivery (mmHg)	112 ± 7	143 ± 5	< 0.001*	
Diastolic blood pressure at delivery (mmHg)	66 ± 5	94 ± 6	< 0.001*	
Sex of newborn (n of male/female)	964/852	108/115	0.025^**†**^	
Birth weight (g)	3096 ± 549	2791 ± 462	< 0.001*	
Preeclampsia (percentile)		2511± 382 (40)		
Gestational hypertension (percentile)		2957± 413 (50)		
Hypertensive disorder in pregnancy	112 (6.2)	37 (16.6)	< 0.001^**†**^	3.0 (2.0–4.5)
Preeclampsia	48 (2.6)	13 (5.8)	< 0.001^**†**^	2.3 (1.2–4.3)
Gestational hypertension	64 (3.5)	24 (10.8)	< 0.001^**†**^	3.3 (2.0–5.4)
Aspirin treatment	None	None		
Smoking	None	None		

* Data: mean ± SD, p-Value: <0.05 via Student’s t-test

^**†**^ Data: n (percent), p-Value: <0.05 via χ^2^ test

Of the 134 women whose BP was measured at least twice, 31 were later diagnosed with HDP (22.1%) (Table 2). Initial BP measurement was performed at a mean of 8.3 gestational weeks and average duration of measurement was 3 weeks.

**Table 2:** Frequency of the occurrence of hypertensive disorders of pregnancy according to the number of prehypertension measurements at separate hospital visits in pregnant women with prehypertension before 20 weeks of gestation.

	Number of prehypertension measurements at separate visits			
	Once	Twice	Thrice	≥ 4 times
Number of pregnant women	89	106	24	4
Hypertensive disorders of pregnancy				
Preeclampsia	3 (3.4)	7 (6.6)	2 (8.3)	1 (25.0)
Gestational hypertension	3 (3.4)	10 (9.4)	8 (33.3)	3 (75.0)

Data: number (percent)

**Fig 2:**
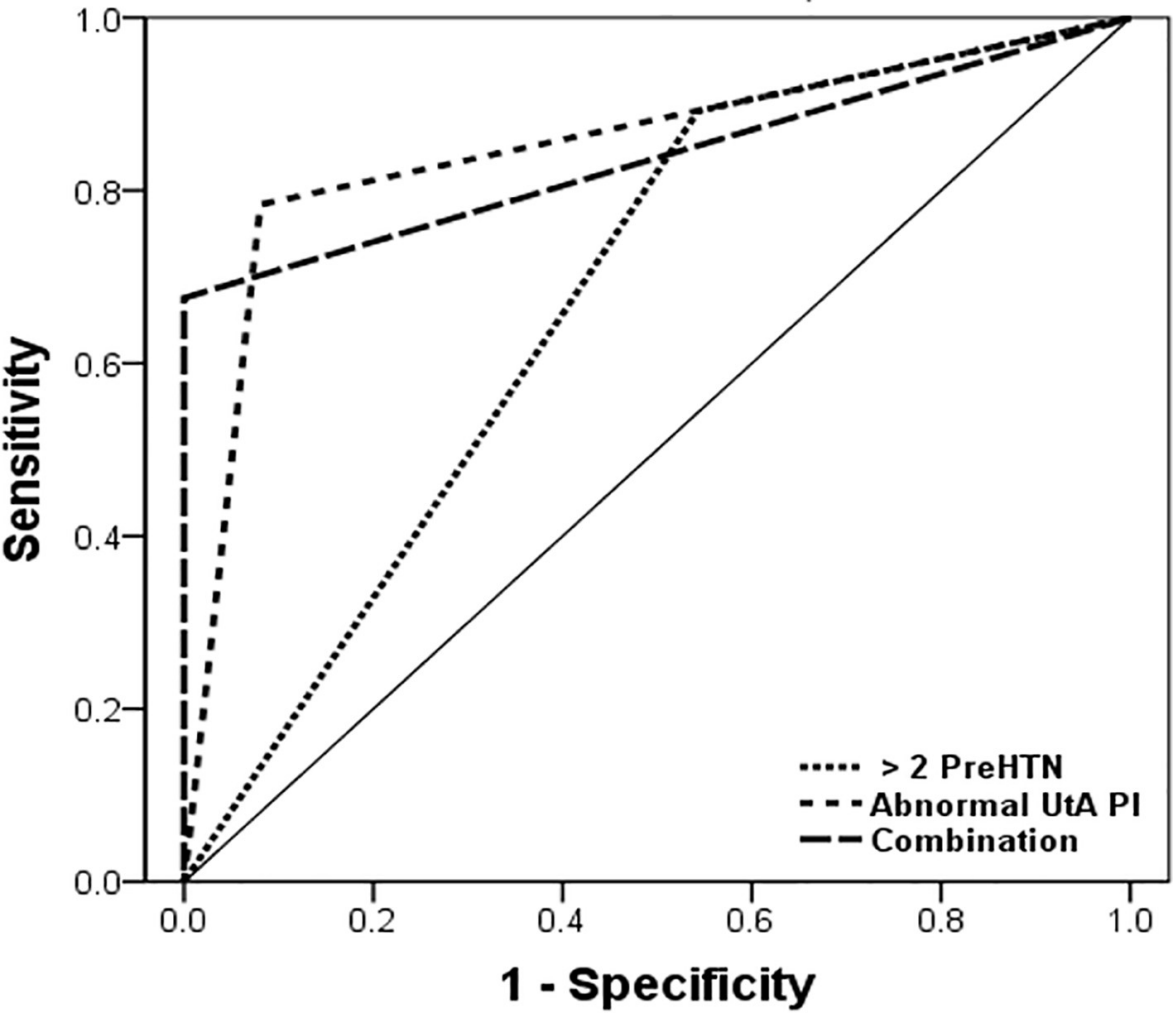
The receiver operating characteristic (ROC) curve of preHTN history and abnormal UtA Doppler for HDP predictions in preHTN group. This curve had the following area under the curve (AUC) values: 0.67 for a history of preHTN, 95% confidence interval (CI) 0.59–0.75; 0.80 for abnormal UtA Doppler, 95% CI 0.77–0.93; and 0.85 for Combination, 95% CI 0.74–0.93.

The performance of two or more measurements of preHTN, abnormal UtA Doppler velocimetry, and their combination in predicting HDP in the preHTN group is are shown in Table 3. The two or more preHTN measurements had the highest sensitivity for predicting PE, GHTN and HDP. However, history of preHTN had the lowest specificity and positive predictive value than of the other tests. Abnormal UtA Doppler had higher specificity than preHTN history. The combination of preHTN history and Doppler had the highest specificity and positive predictive value for predicting HDP. The ROC curve analysis of each test for prediction of HDP in the preHTN group identified the combined test as more effective than others. (Fig 2).

**Table 3:** Predictive capability of twice or more preHTN measured, abnormal findings of uterine artery Doppler velocimetry, and the combination of both parameters in the preHTN group.

Test	Test positive, n (%)	Category	Sensitivity (%)	Specificity (%)	PPV (%)	NPV (%)	OR (95% CI)	p-Value
Two or more preHTN	134 (60.1)	PE	76.9	41.0	27.5	83.6	1.5 (1.2–1.8)	0.015*
		GHTN	87.5	45.5	35.7	86.6	1.6 (1.0–2.2)	0.008*
		HDP	83.8	47.2	41.1	88.3	1.9 (1.0–3.1)	< 0.001*
Abnormal UtA Doppler	46 (20.6)	PE	69.2	82.4	39.8	71.3	2.1 (1.1–2.9)	< 0.001*
		GHTN	83.3	86.9	40.1	74.9	2.0 (1.6–2.8)	< 0.001*
		HDP	83.8	91.9	49.1	79.5	2.3 (1.6–3.4)	< 0.001*
Two or more preHTN + abnormal UtA Doppler	22 (9.8)	PE	61.5	93.3	62.0	81.3	2.6 (1.5–3.2)	< 0.001*
		GHTN	70.8	97.5	63.8	81.4	2.4 (1.2–4.1)	< 0.001*
		HDP	67.6	98.4	67.5	88.3	2.9 (1.1–4.1)	< 0.001*

Data: n (percent), odds ratios, and confidence interval (CI) * p-Value: <0.05 via logistic regression analysis.

UtA: uterine artery Doppler, PE: preeclampsia, GHTN: gestational hypertension, HDP: pregnancy-induced hypertension, PPV: positive predictive value, NPV: negative predictive value, OR: odds ratio.

## Conclusions

Among pregnancy complications, HDP occur in 10% of all pregnancies and is a major cause of maternal fetal morbidity and mortality.[1] PreHTN is not a disease category; rather, it is a designation used to identify patients at risk for HDP, alerting both patients and clinicians.[7] Some researchers reported that preHTN under 20 gestational weeks can be a risk factor for HDP.[8,9,20]

Similar to other publications, the risk for HDP was higher in the preHTN group than that in the normotensive group. Additionally, the more measurements of preHTN increase the sensitivity for predicting HDP in this study. The definition of preHTN has varied in previous studies. Roseny et al. defined their preHTN group as those with preHTN measured at least once before 20 weeks.[9] Meanwhile, Black et al. defined their preHTN group as those whose preHTN was measured at least twice.[20] Despite these difference, both studies showed that preHTN increased the risk of pregnancy related hypertensive disorders as evidenced by the higher OR (4.6 for HDP in Roney et al.’s study and 2.17 for PE in Black et al.’s study). In our study, we defined the preHTN group as patients whose BP was elevated at least once and further divided the group into patients whose preHTN was measured at least twice. The results of our analysis showed that preHTN increased the risk of HDP (OR: 2.3 for PE, 3.3 for GHTN). Moreover, the risk is significantly increased with repeated preHTN measurements (OR: 1.5 for PE, 1.6 for GHTN, 1.9 for HDP). The combination of both a repeated history of preHTN and abnormal UtA Doppler findings had a higher specificity for predicting the adverse outcomes of PE and GHTN than preHTN history or Doppler alone. These results indicate that multiple measurements of BP is advised in preHTN preganant women. Additionally, UtA Doppler velocimetry is needed in repeated occurrences of preHTN because the combination of preHTN history and UtA Doppler findings has high accuracy for predicting the risk for HDP.

Doppler ultrasonography is generally regarded as a valuable tool for assessing fetal well-being in high-risk pregnancies.[21,22] The presence of an increased uterine artery PI with early diastolic notch suggests increased peripheral vascular resistance and impaired placental perfusion, which may lead to adverse pregnancy outcomes.[18,23] Prediction of PE using Doppler depends on the gestational weeks, and Antsaklis et al. demonstrated that 24 of gestational weeks Doppler value have high sensitivity (76.1%), specificity (95.1%) and positive predictive value (34%).[24] Our study found lower Doppler performance for predicting of PE which may be due to the early weeks of gestation 21 at which studies were performed. For detailed evaluation, further Doppler studies should be done at 24 weeks or more gestational weeks.

Other studies have shown that combine Doppler findings with other clinical parameters to improve their accuracy. Onwudiwe et al. showed that the combination of maternal demographic characteristics, UtA Doppler findings, and maternal BP measurement is an effective screening tool for predicting PE.[16] Caradeux et al. confirmed the advantage of combining multiple clinical variables for prediction of early onset PE during early gestation.[25] To our knowledge, this is the first study to report that preHTN history combined with abnormal UtA Doppler velocimetry findings at <20 gestational weeks indicates a high risk for HDP.

This study has several limitations because of its retrospective observational design. Importantly, although second trimester abnormal Doppler suggests placental function and fetal growth, the lack of serial Doppler measurements data from 20 weeks until delivery is a considerable limitation. In addition, the small number of IUGR cases makes it difficult to assess role of uterine artery Doppler evaluation for fetal growth [17,18] and should be improved by further prospective, multi-center studies Also, because of physiological decreases in BP during the early stage of pregnancy, some instances of preHTN may have been missed.[26–28] Because of the single-center setting, the sample size is limited. However, using such a setting ensured that a standardized process was used for BP and Doppler measurements. Further studies are required to help improve the predictive accuracy of the tests including second trimester Doppler values and measurements of angiogenic factors.

In conclusion, close monitoring and recording of every preHTN event in pregnant women with preHTN are important. Moreover, UtA Doppler velocimetry needs to be performed during the 2^nd^ trimester to predict HDP. In addition, healthcare providers should closely monitor and counsel their patients with preHTN, even in isolated cases.
